# Mapping the landscape of cell type-dependent genetic regulation of DNA methylation across human tissues

**DOI:** 10.21203/rs.3.rs-7895550/v1

**Published:** 2025-11-07

**Authors:** James L. Li, Niyati Jain, Jason Tham Han Kiat, Lin Tong, Meritxell Oliva, Kathryn Demanelis, Jasmine Farzana, Muhammad G. Kibriya, Habibul Ahsan, Lin S. Chen, Andrew E. Teschendorff, Brandon L. Pierce

**Affiliations:** 1 University of Chicago, Public Health Sciences, Chicago, IL, 60637; 2 University of Chicago, Interdisciplinary Scientist Training Program, Chicago, IL, 60637; 3 University of Chicago, Committee on Genetics, Genomics, Systems Biology, Chicago, IL, 60637; 4 Shanghai Institute of Nutrition and Health, University of Chinese Academy of Sciences, Chinese Academy of Sciences, 320 Yue Yang Road, Shanghai 200031; 5 Genomics Research Center, AbbVie, North Chicago, IL 60064; 6 University of Pittsburgh, Department of Medicine, Pittsburgh, PA, 15261; 7 University of Chicago, Department of Human Genetics, Chicago, IL, 60637; 8 University of Chicago, Comprehensive Cancer Center, Chicago, IL, 60637; 9 University of Chicago, Department of Medicine, Chicago, IL, 60637

## Abstract

DNA methylation (DNAm) is an epigenetic modification involved in gene regulation. DNAm quantitative trait loci (mQTLs) have been identified in many tissues, but bulk-tissue studies obscure cell type-specific effects. Here, we present the first multi-tissue landscape of cell type-dependent regulation of DNAm in humans by mapping cell type-interaction mQTLs (imQTLs) across seven tissue types (breast, colon, lung, ovary, prostate, kidney, and whole blood), identifying 3,150 imQTLs. Inter-individual variability in cell type proportion, rather than mean proportion, was most associated with imQTL discovery. The cell type with the most imQTLs tended to have interaction effects directionally consistent with mQTL marginal effects from bulk-tissue. imQTLs exhibited biologically relevant effect sharing across related cell types. Compared to cell-agnostic mQTLs, imQTLs exhibited stronger enrichment in regulatory elements and higher colocalization with eQTLs and GWAS loci. Our cell type deconvolution-based approach provides a scalable alternative to single-cell DNAm profiling for uncovering the cellular contexts of genetic regulation of DNAm.

## INTRODUCTION

DNA methylation (DNAm) is one of the most extensively studied epigenetic modifications in humans and is involved in regulation of gene expression, transcription factor binding, and chromatin structure.^1–3^ Inherited genetic variants that influence DNAm levels, known as DNAm quantitative trait loci (mQTLs), have been identified across multiple human tissue types and provide insight into the etiology of complex traits and diseases.^4^ Prior mQTL studies have predominantly been conducted using bulk-tissue DNAm data consisting of an aggregate of different cell types;^4,5^ as such, genetic effects on DNAm that are cell type-dependent may be obscured or undetectable, especially for effects present in less common cell types within a tissue. Although single-cell DNAm profiling can theoretically resolve cell type-specific mQTLs, widespread adoption of this approach is challenging, in terms of cost and scalability, thus limiting the generation of large-scale datasets.^6–9^ While a few recent studies have utilized genotype-by-cell type interaction models to identify mQTLs with cell type-dependent effects in whole blood,^10,11^ a comprehensive map of cell type-dependent genetic regulation of DNAm across diverse human tissues has yet to be established.

To address this gap, we mapped cell type-interaction mQTLs (imQTLs) across seven tissue type including colon, lung, ovary, prostate, breast, kidney, and whole blood by integrating *in silico* estimates of cell type proportions obtained from deconvoluting bulk-tissue DNAm data. After mapping imQTLs, we explored the behavior of genotype-by-cell type interaction terms across various cell and tissue types and evaluated their colocalization with expression quantitative trait loci (eQTLs) and GWAS of health-related traits and diseases. This study provides the first map of imQTLs across multiple human tissue types, revealing cell type-dependent genetic effects on DNAm.

## RESULTS

### Sample characteristics:

This study included whole blood DNAm and genotyping data from 1,182 participants in the Health Effects of Arsenic Longitudinal Study (HEALS),^12^ as well as paired tissue-specific DNAm and whole genome sequencing data from 363 donors from the Genotype-Tissue Expression (GTEx) project^4,13,14^. DNAm data from GTEx included seven tissue types (breast, lung, colon, ovary, prostate, kidney, and whole blood), for which DNAm-based cell type deconvolution panels existed (as described below), out of nine total GTEx tissue types previously profiled for DNAm. DNAm sample sizes for each GTEx tissue type ranged from 39 to 190 (**Supplementary Table 1**). Participants from HEALS were Bangladeshi adults, with an average age of 38.7 years. GTEx donors were primarily of European ancestry, with mean ages ranging from 49.9 to 59.4 years across tissue types (**Supplementary Table 1**). A higher proportion of GTEx donors were ever smokers (64.8% to 78.8% across tissues), in comparison to 43.6% in HEALS (**Supplementary Table 1**). In all analyses, whole blood DNAm data from HEALS and GTEx were analyzed independently.

### Cell types inferred from bulk DNAm data:

**Figure 1.**
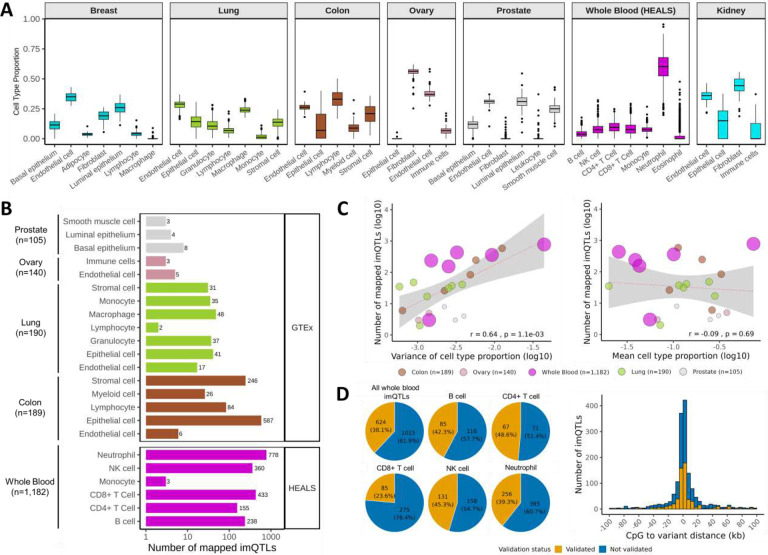
Discovery and validation of cell type-interaction mQTLs (imQTLs) across diverse human tissue types. A) Cell type proportions inferred in each tissue type using EPISCORE. Cell type proportions presented for whole blood are from HEALS samples. B) Number of imQTLs identified for each cell type/tissue combination at a Bonferroni-adjusted-p-value<0.05. These imQTL counts are defined by the number of unique CpG-variant pairs for a given cell-tissue type combination. C) Scatterplots of number of mapped imQTLs vs. variance of cell type proportions (left) and vs. mean cell type proportions (right). Sizes of dots correspond to the sample size of each tissue used to map imQTLs. D) Validation of whole blood imQTLs mapped in HEALS samples in GTEx samples including the proportion of imQTLs that validated by cell type (left) and the distance from CpGs to lead variants for imQTLs color-coded by validation status in a 200 kb window (right). Note: the pie chart for monocytes is not presented as only two imQTLs were tested for validation (1 validated, 1 did not validate)

Prior to mapping cell type-interaction mQTLs, we generated *in silico* estimates of cell type proportions from bulk-tissue DNAm data for the aforementioned 7 tissue types using two reference-based deconvolution methods: “EpiDISH” (v2.14.1) and “EpiSCORE” (v0.9.5).^15,16^ Since a DNAm-based cell type deconvolution panel did not exist for non-diseased ovarian tissue, we developed a generic four-cell type panel (*centEpiFibEndoIC.m*) to deconvolute epithelial cells, endothelial cells, fibroblasts, and immune cells; this panel validated robustly across all four cell types in simulated *in silico* mixtures (**Supplementary Figure 1**). When we utilized this generic panel to deconvolute ovary samples in our study, we observed that fibroblasts emerged as the major cell type while endothelial cells, immune cells, and epithelial cells were minor cell types (Figure 1). This distribution was largely consistent with cell type proportions previously inferred from single-cell RNA sequencing of an independent normal ovary sample not included in our study, in which fibroblasts were also estimated to be the predominant cell type.^17^ To further demonstrate the utility of this generic four-cell panel for deconvoluting epithelial tissues, we applied this panel to colon and lung tissues in our study and observed high concordance in relative cell type proportions with those estimated using existing tissue-specific deconvolution panels (**Supplementary Figure 2, Supplementary Figure 3**).

Across the 7 tissue types, we deconvoluted a total of 40 cell-tissue type combinations (Figure 1, **Supplementary Figure 4**) and found that in several tissues including lung and colon, immune cell types were highly correlated with other immune cell types, and non-immune cell types were similarly correlated among themselves (**Supplementary Figure 5**). Among the cell types estimated, some showed large proportions of individuals (>50%) whose estimated proportion for that cell type was 0 – such as macrophages in breast, epithelial cells in ovary, fibroblasts and leukocytes in prostate, immune cells in kidney, and eosinophils in whole blood – and were therefore excluded. The inferred cell type proportions were generally correlated with tissue-specific principal components (PCs) generated from DNAm data using the “PCAforQTL” (v0.1.0) package^18^ (**Supplementary Figure 6**); these DNAm-derived PCs were utilized to control for potential batch effects.

### Mapping cell type-interaction mQTLs (imQTLs) across diverse tissue types:

To identify cis-mQTLs whose effects varied with the inferred proportion of a given cell type, we tested genotype-by-cell type interactions in linear regression models with DNA methylation as the outcome, adjusting for age, sex, the top 5 genetic PCs, and an optimal number of DNAm-derived PCs selected by “PCAforQTL” for each tissue type (further detailed in the Online Methods). We refer to these mQTLs as cell type-interaction mQTLs (imQTLs), defined as unique lead variant-CpG pairs within a given cell and tissue type combination. In total, we identified 3,150 imQTLs across five tissue types (i.e., the tissues with the highest sample sizes, including whole blood samples from HEALS and colon, lung, ovary, and prostate samples from GTEx) after correcting for multiple testing (Bonferroni-adjusted p-value<0.05) (Figure 1B, **Supplementary Table 2**). These 3,150 imQTLs corresponded to 1,981 unique lead variants and 2,343 unique CpGs. In general, we observed that the number of imQTLs detected increased with increasing sample size (Figure 1B). For tissue types with sample sizes <50, including kidney, breast, and whole blood samples from GTEx, zero imQTLs were detected; thus, subsequent analyses of whole blood imQTLs are based on those mapped in HEALS samples. The interaction terms from genome-wide cis-imQTL analyses exhibited little evidence of systematic inflation (**Supplementary Figure 7**). To serve as a comparison, we mapped mQTLs using the same samples as for imQTL analyses and observed a similar trend that increasing sample sizes were correlated with an increasing number of detected mQTLs (as described previously^4^), with the number of detected mQTLs ranging from 2,882 in breast to 250,909 in whole blood (**Supplementary Figure 8**). Of note, imQTL analyses identified additional CpG sites not identified based on traditional mQTL mapping in matching tissues, including 80 CpG sites in colon, 8 in lung, 5 in whole blood, and 2 in ovary (**Supplementary Table 3**).

Within each tissue type, we observed that cell types with a larger variance in their inferred proportions across individuals had larger numbers of detected imQTLs compared to cell types with lower variance (Figure 1C); in contrast, the mean inferred cell type proportions were less indicative of power to map imQTLs (Figure 1C). The lead variant and target CpGs for most imQTLs were in close proximity, with a mean distance of 19.4 kb (standard deviation [SD]: 45.1 kb), which was lower than the mean distance of 39.8 kb (SD: 79.9) for mQTLs mapped in matching tissues (**Supplementary Figure 9**). We observed that many imQTL variants impacted multiple CpGs, with an average of 1.9 CpGs per variant (SD: 2.1), and a maximum of 25 CpGs regulated by a single variant (**Supplementary Figure 10**). We performed conditional analyses within 500 kb of each imQTL’s lead variant and identified 106 imQTLs with an independent secondary signal nearby, most of which (40.6%) were identified for neutrophils in whole blood (**Supplementary Figure 11**).

### Validation of imQTLs mapped in whole blood:

Given that our primary analysis included imQTLs mapped in whole blood samples from HEALS, we performed validation of these imQTLs in GTEx whole blood samples. We observed that the interaction term estimates across all HEALS imQTLs showed clear correlation with interaction terms computed in GTEx samples (r^2^=0.56) (**Supplementary Figure 12A**), with strong correlation (r^2^=0.81) when restricting to GTEx imQTLs with a nominal p-value<0.05 (**Supplementary Figure 12B**). When utilizing a nominal p-value threshold<0.05 and same sign of interaction terms as criteria for validation, we observed 38.1% of whole blood imQTLs validated in GTEx across all cell types, with up to 48.6% for CD4+ T cells (Figure 1D), highlighting the very limited sample sizes for imQTL analyses in GTEx whole blood. Distances between imQTL lead variants and target CpGs were similar between imQTLs that validated (mean: 18.4 kb, SD: 37.8 kb) and those that did not validate (mean: 20.5 kb, SD: 40.4 kb) (Figure 1D).

### Examining interaction terms of imQTLs in context of marginal and main “effects”:

**Figure 2.**
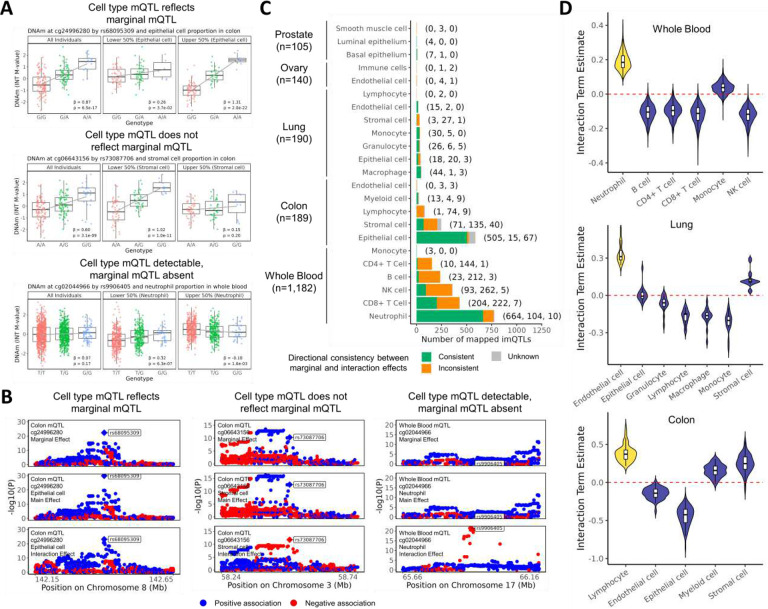
Examining interaction, main, and marginal effects of imQTLs. A) Example methylation vs. genotype boxplots of imQTL classes defined by directional consistency between marginal and interaction effects. Lead variant is labeled and indicated with a diamond shape. B) Example regional plots of imQTL classes defined by directional consistency between marginal and interaction effects. C) Bar plot of the number of imQTLs with consistent, inconsistent, and unknown directional consistency between marginal and interaction effects. Unknown variants were defined as those with a marginal effect p-value>1×10^−5^. Format of parenthesis to the right of each bar is as follows: (number consistent, number inconsistent, number unknown). D) Violin plots of the relative sign of interaction terms of CpG-variant pairs mapped for imQTLs identified for the major cell type of each tissue. The major cell types of whole blood, lung, and colon are neutrophils, endothelial cells, and lymphocytes, respectively. To facilitate comparison across cell types, the interaction terms of major cell types have been set to positive.

Given that imQTLs represent mQTLs whose effects differ by cell type proportion, we examined the directional consistency of imQTL interaction terms with their corresponding genotype effects on DNAm estimated from cis-mQTL models without interaction terms (hereafter referred to as the “marginal effect”). Based on directional consistency between interaction terms and marginal association estimates, we propose three classes of imQTLs. First, imQTLs with consistent directionality represent cases where the cell type mQTL reflects the marginal mQTL (Figure 2A **top**, Figure 2B **left**); these mQTLs represent cases where the modeled cell type (i.e. the cell type included in the interaction model) contains a genetic effect that is detectable at the bulk-tissue level (or where several cell types including the modeled cell type exhibit a similar a genetic effect). Second, imQTLs with inconsistent directionality represent cases where the cell type mQTL does not reflect the marginal/bulk mQTL (Figure 2A **middle**, Figure 2B **middle**); this class consists of mQTLs that are present in other cell types but not in the modeled cell type, as well as mQTLs where the modeled cell type exhibits a cell type-specific genetic regulatory effect in an opposite direction to that observed in other cell types. The third class consists of imQTLs where the marginal effect is undetectable but the cell type mQTL is detectable as an interaction (Figure 2A **bottom**, Figure 2B **right**); this class consists of mQTLs that are specific to the modeled cell type (likely a minor cell type) and either absent or showing an opposite direction of effect in other cell types. Based on these classes, across most tissues, we found that imQTLs mapped to the cell type with the highest number of detected imQTLs were generally of the first imQTL class, showing interaction effects that were directionally consistent with the marginal mQTL effects. These cell types include neutrophils in whole blood (85.3% directionally consistent), epithelial cells in colon (86.0%), macrophages in lung (91.7%), and basal epithelium in prostate (87.5%). imQTLs mapped to endothelial cells in ovary did not follow this pattern and were mostly of the second imQTL class and were directionally inconsistent (20.0% consistent) (Figure 2C). Notably, major cell types (based on abundance) did not always tend to show directional consistency, including colon lymphocytes, for which imQTLs were mostly directionally inconsistent with marginal associations (1.2% consistent).

We next examined how the interaction terms for imQTLs mapped to major cell types varied across other cell types within the same tissue. We restricted this analysis to whole blood, lung, and colon, where a substantial number of imQTLs were detected. For whole blood and lung, most minor cell types showed interaction terms with signs opposite to that of the major cell type (Figure 2D). This sign reversal was primarily observed for minor cell types whose proportions were negatively correlated with the major cell type (**Supplementary Figure 5**). Additionally, we examined whether main effects of imQTLs in models that included interaction terms systemically differed from marginal mQTL effects (from models with no interaction term), but we did not observe any consistent patterns of attenuation or strengthening of main effects in comparison to marginal effects across cell-tissue type combinations (**Supplementary Figure 13**).

### Sharing of imQTL interaction effects across cell types:

**Figure 3.**
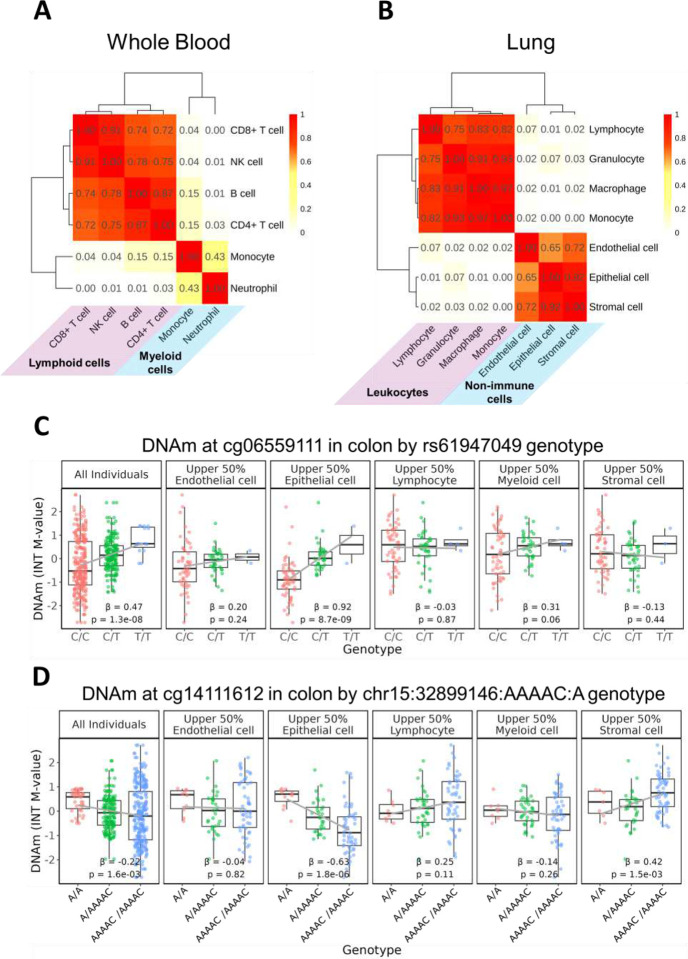
Evaluating cell type sharing and heterogeneity of imQTLs. A) Proportion of shared effects for imQTL interaction terms across cell types in whole blood. B) Proportion of shared effects for imQTL interaction terms across cell types in lung. C) Example of a candidate cell type-specific imQTL in colon epithelial cells where an mQTL effect is only present in one cell type; i.e. no other cell types showed a nominally significant interaction (p<0.05) and the genotype-methylation association was nominally significant (p<0.05) among individuals in the upper 50% for that cell type’s estimated proportion, but not in individuals enriched for other cell types. D) Example of a candidate cell type-specific imQTL where two cell types in colon (epithelial and stromal cells) demonstrate cell type-specific mQTL effects.

We assessed the proportion of shared effects between cell types within whole blood, lung, and colon by computing the proportion of interaction terms that had a consistent sign and nominal p-value<0.05 amongst all imQTLs identified for a given tissue type. We found that in whole blood, cells arising from a lymphoid progenitor (CD8+ T cell, CD4+ T cell, NK cell, and B cell) tended to cluster together while those arising from a myeloid progenitor (neutrophil and monocyte) also had a high proportion of shared effects (Figure 3A). In lung, we observed clustering of effects among immune cells (lymphocyte, granulocyte, macrophage, and monocyte) and non-immune cells (endothelial cell, epithelial cell, stromal cell) (Figure 3B). On the other hand, we did not observe biologically informative clustering of cell types in colon (**Supplementary Figure 14**). In general, the clustering of these cell types reflected the correlations between cell type proportions among individuals (**Supplementary Figure 5**), suggesting these correlations may contribute to imQTL effect sharing observed.

### Identifying candidate cell type-specific imQTLs:

We next identified candidate cell type-specific imQTLs, defined as those with interaction effects that were specific to a single cell type. In particular, we considered an imQTL to be cell type-specific if (1) the interaction term for the modeled cell type had the opposite sign compared to other cell types or if no other cell types showed a nominally significant interaction (p<0.05), and (2) the genotype-methylation association was nominally significant (p<0.05) among individuals in the upper 50% for that cell type’s estimated proportion, but not in individuals enriched for other cell types unless the association was of opposite directionality. Using these criteria, we identified a total of 30 candidate cell type-specific imQTLs including 24 mapped to epithelial cells in colon, 1 to myeloid cells in colon, 3 to stromal cells in colon, and 2 to neutrophils in whole blood (**Supplementary Table 4**). An example of a cell type-specific imQTL is rs61947049 at cg06559111 in colon where the marginal association observed across all individuals appears to be predominantly driven by the association in epithelial cells (Figure 3C). In contrast, the variant chr15:32899146:AAAAC:A at cg14111612 was identified as having a cell type-specific interaction term separately for epithelial and stromal cells, and the association in the upper 50% of individuals enriched for each cell type showed opposite directionality (Figure 3D).

Although a single-cell mQTL database is not currently available to validate these candidate cell type-specific imQTLs, we instead queried them in scQTLbase,^19^ one of the largest collections of human single-cell eQTLs comprised of over 300 datasets; these eQTLs primarily were mapped in various white blood cell and neural cell types. Among the 29 candidate cell type-specific imQTLs, 12 had been previously identified as cell type-specific eQTLs (**Supplementary Table 5**).^20–23^ Notably, of the 24 cell type-specific imQTLs identified in colon epithelial cells, 4 were also found to be cell type-specific eQTLs in floor plate progenitors,^20^ which give rise to specialized epithelial cells forming the ventral midline of the neural tube.^24^

### Enrichment of target CpGs in site differentially methylated by cell type:

**Figure 4.**
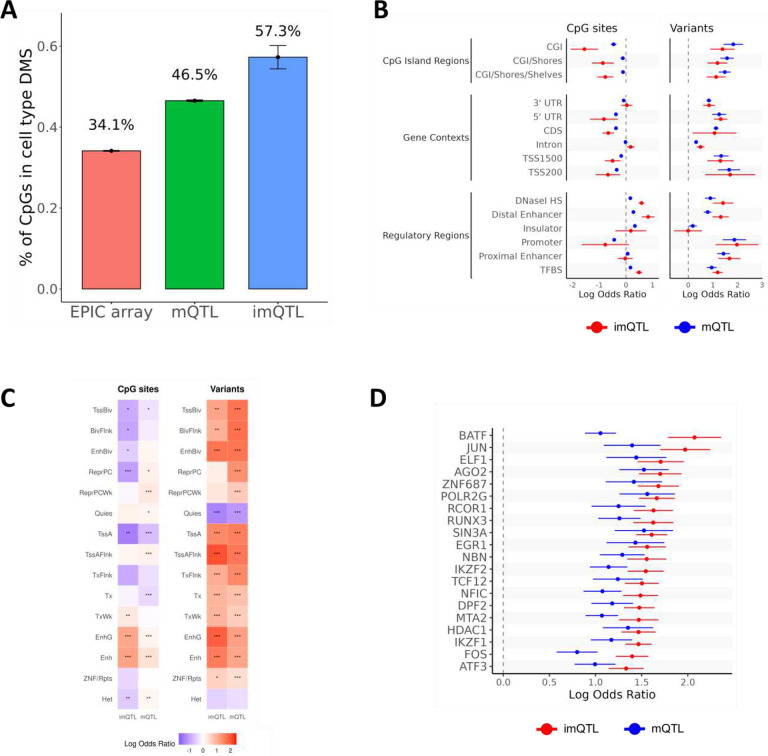
Enrichment of imQTLs for genomic context. A) Enrichment of whole blood imQTLs identified in HEALS in differentially methylated sites (DMS) by cell type identified in GTEx whole blood samples. B) Enrichment of whole blood, colon, and lung imQTLs in CpG islands, gene contexts, and regulatory regions. C) Enrichment of whole blood, colon, and lung imQTLs in tissue-specific chromatin segmentation states. * p<0.05, ** p<0.01, *** p<0.001. D) Enrichment of whole blood, colon, and lung imQTLs in TFBS binding sites. Note: The displayed log odds ratios were obtained using a random-effects meta-analysis of associations in whole blood, colon, and lung. The top 20 enriched TFBS for imQTLs are shown.

We explored whether whole blood imQTLs identified in HEALS were enriched in differentially methylated sites (DMS) by cell types; these DMS were identified by performing an epigenome-wide association study for each cell type in GTEx whole blood samples that tested the association between proportions of each cell type and every EPIC array CpG site. For reference, we observed that 34.1% of all EPIC array CpGs were in cell type DMS (Figure 4A). The proportion of CpGs mapped to whole blood imQTLs in DMS was 57.3%, even higher than the 46.5% of whole blood mQTLs that were cell type DMS (Figure 4A).

### Enrichment of lead imQTL variants and target CpGs in islands and regulatory features:

We evaluated enrichment of imQTLs in whole blood, colon, and lung in regulatory elements and observed that lead variants were generally enriched in CpG islands (CGIs), regions near transcribed genes, and regulatory regions including DNase I hypersensitivity sites, enhancers, promoters, and transcription factor binding sites (TFBS) (Figure 4B, **Supplementary Figure 15, Supplementary Table 6**). In contrast, CpGs impacted by imQTLs were depleted in CGIs, regions upstream of transcribed genes, 5’ UTRs, coding sequences, and promoters, but were enriched for DNase I hypersensitivity sites, enhancers, and TFBS (Figure 4B, **Supplementary Figure 15, Supplementary Table 6**). When examining enrichment for tissue-specific chromatin segmentation states catalogued by the NIH Roadmap Epigenomics Mapping Consortium,^25^ we observed enrichment for both imQTL lead variants and CpG sites in enhancers; however, lead variants were also enriched in chromatin states related to transcription, while CpGs were depleted in such states (Figure 4B, **Supplementary Figure 16, Supplementary Table 7**). In general, enrichment of mQTLs in these regulatory elements was similar to imQTLs, but enrichment of both imQTL variants and CpGs in DNase I hypersensitivity sites and enhancers were markedly higher than for mQTLs, and the depletion of mapped CpGs in CGI was more pronounced for imQTLs (Figure 4B&C).

We assessed enrichment of imQTL lead variants in specific TFBS and identified POLR2A as the most enriched TFBS, separately in colon, lung, and blood, which encodes for the largest subunit of RNA polymerase II^26^ (**Supplementary Figure 17, Supplementary Table 8**). IKZF1, which is essential for development of lymphoid lineages,^27^ emerged as the strongest signal in a random-effects meta-analyses across all three tissues, driven primarily by its enrichment among whole blood imQTLs (Figure 4D, **Supplementary Table 8**). Interestingly, we observed enrichment for cancer-related transcription factors including MYC and JUN family members in all three tissue-specific analyses, as well as in the meta-analysis (**Supplementary Table 8**).

### Colocalization of imQTLs with eQTLs and GWAS traits and diseases:

**Figure 5.**
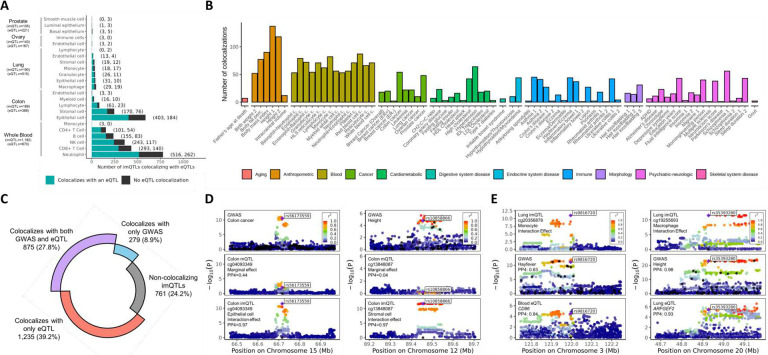
Colocalization with eQTLs and GWAS traits. A) Number of imQTLs that colocalized with eQTLs. Sample sizes utilized in the mapping of imQTLs and eQTLs are listed in the parentheses under each tissue label. B) Number of colocalizing imQTLs by GWAS trait. C) Arc chart of the proportion of imQTLs colocalizing with eQTLs and GWAS traits. D) Example regional plots for imQTLs where colocalization with GWAS traits was detected using interaction terms but not marginal effects. E) Example regional plots for imQTLs that colocalized with both eQTLs and GWAS traits. Note: PP4 values listed correspond to colocalization of genetic associations between the present panel and the top panel. Default priors in the coloc R package were utilized including p_1_=1×10^−4^, p_2_=1×10^−4^, and p_12_=1×10^−5^.

For imQTLs detected in five tissues (whole blood, colon, lung, ovary, prostate), we performed colocalization analyses of imQTL interaction terms with expression quantitative trait loci (eQTLs) obtained from matching tissues in GTEx (v8) using the “coloc” R package (v5.2.3)^28^ with default priors (p_1_=1×10^−4^, p_2_=1×10^−4^, and p_12_=1×10^−5^). We observed evidence of colocalization with an eQTL in at least one tissue (posterior probability of colocalization, PP4>0.5) for 67.0% of all imQTLs (Figure 5A&C, **Supplementary Figure 18A, Supplementary Table 9, Supplementary Figure 19**). When instead utilizing biologically informed priors described in the Online Methods (p_1_=5×10^−4^, p_2_=4.5×10^−3^, and p_12_=5×10^−4^), we observed that up to 83.9% of imQTLs colocalized with an eQTL in at least one tissue (**Supplementary Figure 18B**). The proportion of imQTLs that colocalized with eQTLs in matching tissues ranged from 13.3% in prostate to 42.5% in colon (**Supplementary Figure 18A**).

We also examined colocalization between imQTL interaction terms and GWAS association signals for 90 health-related traits and diseases^29–51^ (**Supplementary Table 10**) and identified 36.6% of imQTLs colocalizing with GWAS signals across a variety of health categories (PP4>0.5) when utilizing default coloc priors^28^ (Figure 5C, **Supplementary Figure 20A, Supplementary Figure 21, Supplementary Table 11**), and up to 63.4% of imQTLs when utilizing biologically informed priors described in the Online Methods (p_1_=1.5×10^−3^, p_2_=3.5×10^−3^, and p_12_=1.5×10^−3^) (**Supplementary Figure 20B**). Traits with the highest numbers of colocalizations included anthropometric traits such as height and weight, cardiometabolic traits, and blood-related traits including blood cell counts, which may reflect the large sample sizes utilized in those GWAS, as well as the high abundance of imQTLs mapped in whole blood (Figure 5B). Interestingly, we also observed a large number of colocalizations for cancers at various body sites such as colon and prostate cancer, including imQTLs that were mapped in the corresponding tissues (Figure 5B, **Supplementary Table 11**). Among the 36.6% of imQTLs demonstrating evidence for colocalization with GWAS, most colocalized with only one GWAS trait/disease; however, imQTLs colocalized with many GWAS traits included chr17:46018101:T:C (rs58879558) –cg19832721, mapping to neutrophils in whole blood, that colocalized with 23 GWAS traits/diseases including ovarian cancer, blood cell counts, Crohn’s disease, lupus, sleep duration, hypertension, hair loss, and height (**Supplementary Table 11, Supplementary Figure 22**). This imQTL also regulated cg23519755 and colocalized with an eQTL for the pseudogene *LRRC37A4P* in all five eQTL tissues examined (PP4=1.00) (**Supplementary Table 9**).

We evaluated the effectiveness of utilizing interaction terms versus marginal effects of these imQTLs in assessing colocalization with GWAS. In total, we identified 2,590 GWAS-colocalizing pairs when using interaction terms, 2,384 when using marginal effects, and 2,177 that were shared between both approaches (**Supplementary Figure 23A**). Among these shared pairs, 53.4% exhibited a higher PP4 when interaction terms were used. At the imQTL level, 56.2% demonstrated increased PP4 values when utilizing interaction terms, and an additional 10.5% of imQTLs were uniquely identified as colocalizing with GWAS when using interaction terms that were missed when utilizing marginal effects (**Supplementary Figure 23B**). For example, rs56173559 was identified as an imQTL regulating cg04093349 in colon epithelial cells and showed colocalization with a colon cancer GWAS signal when interaction terms were utilized, but not marginal effects (Figure 5D **left**). Consistent with this finding, the association between DNAm and genotype was more pronounced among individuals with high epithelial cell proportions (upper 50%) compared to the marginal association observed across all individuals (**Supplementary Figure 24A**). In another example, rs10858866 was mapped as an imQTL in colon stromal cells affecting DNAm at cg13848087 and similarly exhibited colocalization with GWAS signals for height only when interaction terms were utilized (Figure 5D **right**). For this imQTL, the marginal association between DNAm and genotype was absent, while we observed opposite associations for this imQTL among individuals in the lower and upper 50% of stromal cells (**Supplementary Figure 24B**).

We further observed that 27.8% of imQTLs colocalized with both eQTL and GWAS (Figure 5C). For instance, rs9816720 was mapped as an imQTL regulating DNAm at cg20356878 in lung monocytes – with a more pronounced association in individuals enriched for monocytes (**Supplementary Figure 25A**) – and also colocalized with a previously identified eQTL regulating *CD86* in whole blood, a co-stimulatory molecule commonly expressed on lung monocytes/macrophages that is involved in triggering inflammation and immune responses to allergens (Figure 5E **left**).^52,53^ Notably, this imQTL also colocalized with a GWAS signal for hay fever and allergic rhinitis which are conditions where monocyte recruitment is a hallmark immune response (Figure 5E **left**).^54,55^ Another example of an imQTL that colocalized with both eQTL and GWAS signals was mapped at rs35392380 for its regulation of cg19255693 in lung macrophages (Figure 5E **right**), with a more pronounced association in individuals enriched for macrophages (**Supplementary Figure 25B**). Though common variants in *ARFGEF2* – which plays a role in vesicle trafficking^56,57^ – have been previously implicated in GWAS of height,^58,59^ our study suggests that these variants may influence height through regulation of *ARFGEF2*. Taken together, these results demonstrate that modeling imQTLs may provide increased power to detect mQTLs that drive GWAS associations and help to identify relevant cell types underlying those associations.

## DISCUSSION

In this study, we generated the first comprehensive map of cell type-dependent regulation of DNAm across multiple human tissues by mapping cell type-interaction mQTLs. Using data from >1,000 South Asian participants in HEALS and >300 GTEx multi-tissue donors, we identified >3,000 imQTLs across five tissue types including colon, lung, ovary, prostate, and whole blood. For some tissue types, statistical power for detecting imQTLs was heavily constrained by low sample sizes, with no imQTLs detected in tissues with <50 samples. We found that inter-individual variability in cell type proportion, rather than average abundance of a cell type, was more strongly associated with the abundance of imQTLs identified for any given tissue-cell type combination. imQTLs showed stronger enrichment in regulatory elements such as enhancers and DNase I hypersensitivity sites compared to mQTLs detected by traditional mQTL mapping and exhibited higher rates of colocalization with eQTLs – 67.0% in our analysis versus 21% previously reported for mQTLs mapped using the same GTEx samples analyzed in this study.^4^ We additionally observed a high rate of colocalization with GWAS of health-related traits/diseases (36.6%) and demonstrated that utilizing interaction terms can improve power for colocalization compared to using marginal mQTL association results. Taken together, these findings suggest that imQTLs can capture genetic effects that are more relevant to gene regulation and human health than traditionally mapped mQTLs.

This study significantly advances our understanding of the behavior of genotype-by-cell type interaction effects, particularly in tissues with highly variable cell type composition. While previous studies have utilized bulk-tissue RNA sequencing data to model cell type-interaction eQTLs across multiple tissue types,^60,61^ these studies have typically been limited to one or two deconvoluted cell types per tissue; in contrast, our study examined interaction effects across four to seven deconvoluted cell types per tissue. We observed that imQTLs mapped to major cell types often showed an opposite sign of their interaction terms in minor cell types that were negatively correlated with the major cell type. In general, this correlation among cell type proportions in each tissue underscores a limitation in our imQTL-based approach, in that it is typically not possible to definitively identify the specific cell type(s) responsible for an imQTL signal. The whole blood imQTLs identified in our study, as well as those reported in a prior investigation of imQTLs,^11^ showed marginal effects that tended to have high directional consistency with interaction terms for the major cell type (neutrophils). However, we observed a different pattern in colon: only 1 in 84 imQTLs mapped to lymphocytes (major cell type) showed interactions terms directionally consistent with marginal associations, whereas 505 of 587 imQTLs mapped to epithelial cells (minor cell type) exhibited directional consistency. While marginal effects are generally thought to reflect major cell types due to their higher abundance, the consistent effects observed at imQTLs in minor cell types with high variability suggest that marginal effects can also reflect cell type-specific regulatory variation in these less abundant but well-powered cell types. This notion is further supported by the fact that most of our candidate cell type-specific imQTLs were identified in colon epithelial cells, a minor cell type in colon for which the most imQTLs were detected in this tissue.

Despite mapping numerous imQTLs, our study had several limitations. First, imQTLs that we mapped were limited to cell types present in deconvolution panels. While a generic four-cell type panel allowed to us to deconvolute and map imQTLs in ovarian tissue, this panel lacked several tissue-specific cell types including granulosa cells, theca cells, and smooth muscle cells preventing us from assessing cell type-dependent effects in these cell populations. Second, while we identified a set of high-confidence candidate cell type-specific imQTLs – primarily mapped to colon epithelial cells – we were unable to fully validate these associations due to lack of single-cell DNAm profiling data for colon tissue. However, we observed that lead variants of several candidate cell type-specific imQTLs were also identified as cell type-specific eQTLs in cell types including epithelial cell progenitors, highlighting the potential of imQTL-based studies to reveal sites of cell type-specific genetic regulation. Though well-powered single-cell eQTL and mQTL datasets in colon are currently unavailable, additional validation of these candidate imQTLs in such datasets is warranted. Lastly, although variability in cell type proportions was high in breast and kidney, the sample sizes were insufficient for detecting imQTLs. Future studies with increased sample sizes of DNAm data for these two tissue types will be essential to characterize the cell type-dependent regulatory effects in these tissues.

In summary, our work represents the first multi-tissue map of cell type-dependent genetic regulation of DNAm in humans. By integrating deconvoluted cell type proportions with DNAm and genotype data across diverse tissues, we identified thousands of imQTLs and demonstrated the utility of our approach to reveal regulatory variation missed in bulk-tissue studies. Although single-cell DNAm profiling remains ideal for accurately resolving cell type-specific regulatory effects, its limited scalability underscores deconvolution-based approaches such as the one used in this study as a practical alternative for capturing cell type-dependent regulatory effects using existing bulk DNAm data.^62^ These findings provide a valuable resource for understanding the cellular contexts underlying the genetic regulation of DNAm and for identifying potential mechanisms underlying reported GWAS associations.

## ONLINE METHODS

### Processing of DNA methylation data across seven human tissue types:

DNAm was profiled using the Illumina Infinium MethylationEPIC array for whole blood samples from 1,182 participants in the Health Effects of Arsenic Longitudinal Study (HEALS),^12^ a population-based cohort study in Bangladesh, and for seven tissue types – breast, colon, kidney, lung, ovary, prostate, and whole blood – from postmortem donors in the Genotype-Tissue Expression (GTEx) project (**Supplementary Table 1**);^4,13^ we only included breast tissue samples from GTEx obtained from female donors. In HEALS, DNA was extracted from clotted blood using the Qiagen FlexiGene DNA kit and bisulfite-converted using the Zymo EZ-96 DNA Methylation Kit. In GTEx, DNA was extracted using the Qiagen Gentra Puregene method.^4^ For both cohorts, raw DNAm data were processed using “ChAMP”^63^ where quality control procedures included removal of samples with poor probe detection (detection p-value >0.01 in ≥5% of CpGs), sex mismatches, and samples with excessive missingness (>10% missing CpG probes). CpGs were excluded if they had detection p-values >0.01 in any sample, >5% missing values, bead count <3 in ≥5% of samples, overlapped with common SNPs (minor allele frequency >5%) or were within one base pair of a SNP, were cross-reactive, or mapped to sex chromosomes;^64^ missing DNAm values in HEALS were additionally imputed using the k-nearest neighbors method (k=10). For GTEx, beta values were normalized using the single sample normal-exponential out-of-band (ssnoob) method followed by beta-mixture quantile (BMIQ) normalization^65,66^ while beta values in HEALS were normalized just using the BMIQ approach.^65^ Following this, we converted beta values into M-values and subsequently utilized a rank-based inverse normal transformation.^67^ After quality control and filtering, 721,796 CpGs across 1,182 whole blood samples from HEALS individuals and 753,983 CpGs across GTEx tissues were retained, including 190 lung, 189 colon, 140 ovary, 105 prostate, 47 kidney, 47 whole blood, and 36 breast tissue samples.

### Processing of genetic data in GTEx and HEALS:

As previously described, genetic data in GTEx was generated using whole-genome sequencing with a PCR-free protocol on the Illumina HiSeq 2000 and HiSeq X platforms at the Broad Institute and processed according to standardized pipelines.^14^ Genetic data in HEALS was obtained through genotyping using either the Illumina HumanCyto12 300K array or the Infinium Multi-Ethnic EUR/EAS/SAS 1.4M array, and was processed as previously described.^68^ For GTEx and HEALS separately, we converted VCF files to PLINK (v2.0)^69^ PGEN format and filtered variants to retain only those with no missingness among individuals with DNAm data passing quality control, a Hardy-Weinberg equilibrium p-value<1×10^−6^, and a minor allele frequency (MAF) > 0.1 to reduce the number of false positives when mapping imQTLs.

### Generation of principal components for genetic and DNAm data:

We generated principal components (PCs) for genetic data separately for GTEx and HEALS to account for population stratification by first pruning each genetic dataset using an r^2^ threshold of 0.2 in 500 kb windows. We then used PLINK (v2.0)^69^ to generate principal components for each genetic dataset and retained the top five PCs for use in imQTL mapping. In addition, DNAm PCs were generated separately for each tissue using the “elbow” method from the “PCAforQTL” (v0.1.0)^18^ package to account for batch effects and unmeasured confounders; for whole blood, DNAm PCs were generated separately for HEALS and GTEx samples;^18^ we opted to utilize these PCs generated using “PCAforQTL” (v0.1.0)^18^ instead of PEER factors as this approach has been shown to outperform PEER factor analysis in metrics as previously described.^18^

### Inferring cell type proportions in bulk-tissue DNAm data:

We inferred cell type proportions for each of our tissues utilizing the generating in silico estimates of cell type proportions from bulk-tissue DNAm data in 7 diverse tissue types using “EpiDISH” (v2.14.1)^15^ to deconvolute whole blood samples and the “wRPC” function in “EpiSCORE” (v0.9.5)^16^ to deconvolute non-blood tissues.^16^ Since a deconvolution reference panel was not available for non-diseased ovarian tissue, we constructed and validated a generic reference panel to deconvolute cell types commonly found in ovarian tissue similarly using “EpiSCORE” (v0.9.5) as described in the following subsection.

### Construction and validation of a DNAm deconvolution reference panel for four generic cell types:

We built a generic reference panel to deconvolute epithelial cells, endothelial cells, fibroblasts, and immune cells by following a strategy similar to the one described by Zheng SC et al.^70,71^ Briefly, we used the sorted B-cell, NK-cell, CD4+ and CD8+ T-cell, monocyte, neutrophil and eosinophil Illumina 450k samples from Reinius et al^72^ as representative of immune cells. For epithelial cells, we used Illumina 450k ENCODE data (GSE40699) of 11 different epithelial cell-lines: Hipe (human iris pigment epithelial), Saec (small airway epithelial cell), Haec (Human amniotic epithelial cell), Hre (Human renal epithelial), Hrpe (Human retinal pigment epithelial), Prec (prostate epithelial), Hee (Human esophageal epithelial), Hcpe (Human choroid plexus epithelial), Hnpce (Human non-pigment ciliary epithelial), Hmec (Human mammary epithelial), Hrce (Human renal cortical epithelial). For fibroblasts, we used 7 ENCODE cell-lines (Imr90, Progfib, Ag04449, Ag044505, Ag09319, Bj and Nhdfneo). For endothelial cells, we used 2 vascular endothelial EPIC DNAm samples from Moss et al.^73^ Next, we performed 4 differential DNAm analyses using an empirical Bayes method^74,75^ comparing each cell-type to the other 3, selecting differentially methylated CpG sites (DMCs) based on three criteria: (i) a stringent FDR<0.001, (ii) that the CpG is hypomethylated in the cell-type of interest compared to the other 3 (based on the observation that the most cell-type specific DMCs are always unmethylated in the cell-type of interest^76^) and (iii) that the absolute difference in DNAm between the cell-type of interest and the other 3 is larger than 0.7, which ensures that differences are biological and not related to batch or technical effects (as the latter are <0.2). We aimed to identify around 50–60 high quality DMCs for each cell-type. This was possible using absolute DNAm difference thresholds (condition (iii) above) of 0.9 for immune-cell vs rest, 0.8 for epithelial vs rest, 0.81 for fibroblast vs rest and 0.715 for endothelial vs rest. This resulted in 62, 65, 60 and 66 DMCs highly specific for immune cells, epithelial cells, fibroblasts, and endothelial cells, respectively. The DNAm reference panel over the resulting 253 DMCs (*centEpiFibEndoIC.m*) was built by taking the average DNAm over the samples of a given cell-type. For validation of this reference panel, we used independent Illumina DNAm data as follows: for epithelial cells, we selected 15 epithelial samples from the Moss DNAm-atlas,^73^ which included lung and colon epithelial cells, hepatocytes, acinar and pancreatic ductal cells; for endothelial cells, we used 17 high purity pulmonary endothelial cells from Hautefort et al;^77^ for immune-cells, we used the 417 sorted immune-cells from BLUEPRINT;^78^ for fibroblasts, we used 8 fibroblast/stromal samples from SCM2^79^ (HDF258, HDF51 (x2), CCD1079SK, HPLF260, HDF259, HBdSMC265, MRC5). Validation was performed by generating 500 in-silico mixtures, selecting at random one sample from each cell-type, and mixing them together with randomly chosen weights designed to add to 1. “EpiDISH” (v2.14.1)^15^ with the centroid *centEpiFibEndoIC.m* was then applied to infer the cell-type fractions using the RPC (robust partial correlation) method and 500 maximum iterations. Agreement between estimated and true fractions was assessed using pearson correlation coefficients and root mean square error. While we utilized this DNAm reference panel (*centEpiFibEndoIC.m*) to obtain total epithelial, fibroblast, endothelial and immune-cell fractions in ovary, this panel is suitable for performing association analyses in future studies whilst adjusting for potential variations in these 4 cell-types which could otherwise confound associations. It can broadly be used for solid tissues where the dominant components are epithelial, fibroblast, endothelial and immune-cells and for which alternative DNAm reference panels (e.g. via “EpiSCORE” (v0.9.5)^16^) are not yet available.

### Mapping cell type-interaction cis-mQTLs (imQTLs) and cis-mQTLs to obtain interaction terms, main effects, and marginal effects of variant-CpG pairs:

We modeled cell type-interaction cis-mQTLs (imQTLs) separately for each cell and tissue type combination, as well as study (GTEx and HEALS), using linear regressions; for each CpG site, we regressed DNA methylation levels on: (1) the genotype of a nearby variant (within ±500 kb), (2) the estimated proportion of a given cell type (rank-based inverse normal transformed), (3) an interaction term between the genotype and the estimated proportion of a given cell type (rank-based inverse normal transformed), (4) covariates including age, sex, the top five genetic principal components (PCs), and (5) tissue-specific DNA methylation PCs derived using the “PCAforQTL” (v0.1.0) package. Below is this model where DNAm_*i*_ is the DNAm level, G_*i*_ is genotype dosage for individual *i*, C_*i*_ is the inverse-normal transformed estimated proportion of a specific cell type for individual *i*, G_*i*_ × C_*i*_ is the interaction term between genotype and cell type proportion, PC_gen,*ik*_ is the *k*-th genetic PC for individual *i*, PC_DNAm,*il*_ is the *l*-th DNAm PC derived for the tissue of individual *i*, and *L* is the number of DNAm PCs selected for the current tissue:

$${\text{DNAm}}_{i}=\beta_{0}+\beta_{1}G_{i}+\beta_{2}C_{i}+\beta_{3}\left(G_{i}\times C_{i}\right)+\beta_{4}\;{\text{Age}}_{i}+\beta_{5}\;{\text{Sex}}_{i}+\sum_{k=1}^{5}\gamma_{k}\;{\text{PC}}_{\text{gen},\text{ik}}+\sum_{l=1}^{L}\delta_{l}\;{\text{PC}}_{\text{DNAm},\text{il}}+\varepsilon_{i}$$

Linear models were run using “tensorQTL” (v1.0.10)^80^ and were restricted to variants with a MAF>0.1 in both the upper and lower 50% of individuals for each cell type proportion. To identify significant imQTLs, p-values for interaction terms were first adjusted for the number of variants tested per CpG using efficient multiple-testing (EMT) adjustment^81^ and then subjected to a Bonferroni correction based on the total number of CpGs tested. In addition to interaction terms, we extracted main effects of genotypes from these interaction models implemented by “tensorQTL” (v1.0.10).^80^ We additionally utilized “tensorQTL” (v1.0.10)^80^ to map cis-mQTLs in each tissue type by utilizing a nearly identical procedure to that of mapping imQTLs with the exception that we excluded the interaction terms from the linear regression models and applied a general MAF>0.1 cutoff without regard to cell type proportions. Although the interaction model we implemented to map imQTLs technically represents a marginal interaction model – since each cell type was modeled individually rather than jointly across all cell types^82^ – we refer to the genotype effect on DNAm from these cis-mQTL models as the “marginal effect” for simplicity throughout this study.

### Identification of additional independent at imQTL loci and examining evidence of pleiotropy:

We identified independent secondary signals based on interaction terms by regressing DNAm levels separately on all variants within ±500 kb of each lead imQTL variant, including the lead variant itself. Variants with an interaction p-value<1×10^−5^ were considered candidate secondary signals. Furthermore, for each imQTL lead variant, we examined whether there was evidence of pleiotropy by determining how many CpGs the lead variant was associated with using a significance threshold of p-value<1×10^−4^.

### Enrichment of imQTLs in genomic annotations:

We examined enrichment for genomic annotations of both lead variants and CpGs mapped to imQTLs. For imQTL lead variants, we computed log odds ratios (logORs) and obtained p-values for enrichment by performing Fisher’s exact tests for each genomic annotation compared to the reference of all variants tested within ±500 kb of each CpG; for CpGs, we utilized a similar procedure to obtain logORs and enrichment p-values, but the reference was all available EPIC array CpGs after quality control and filtering for each dataset (GTEx and HEALS). Gene regulatory annotations were retrieved from the ENCODE Encyclopedia version 5 (ENCODE5)^83^ predicted candidate cis-regulatory elements (cCREs) catalog, which include distal enhancers (ENCFF535MKS), proximal enhancers (ENCFF036NSJ), promoter-like elements (ENCFF379UDA), and putative insulators (ENCFF262LCI) (https://www.encodeproject.org/). The classification of putative insulators was defined as CTCF binding sites did not show evidence of overlap with enhancer regions, promoter-like elements, or DNase-H3K4me3 signatures. Gene body annotations were retrieved from GENCODE^84^ release 26 (https://www.gencodegenes.org/human/release_26.html). Chromatin state segmentations based on a 15-state model were obtained from the NIH Roadmap Epigenomics Mapping Consortium^25^ where we utilized the following collections of tissue-specific states: colonic mucosa (E075), lung tissue (E096), and various white blood cells including primary CD4+ T helper naive cells (E038, E039), CD8+ T naive cells (E047), monocytes (E029), B cells (E032), natural killer cells (E046), and neutrophils (E030).

Transcription factor (TF) binding annotations were obtained from ENCODE^83^ versions 2 and 3 and were annotated based on clustered ChIP-seq peaks from 1,256 experiments involving 340 transcription factors across 129 cell and tissue types; this dataset was available on the UCSC Table Browser^85^ (http://genome.ucsc.edu/cgi-bin/hgTables, “encRegTfbsClustered”). CpG island (CGI), shore, and shelf annotations were also retrieved from the UCSC Table Browser^85^ (“cpgIslandExt”), as well as annotations for DNase I hypersensitivity sites (“wgEncodeRegDnaseClustered”) that were derived from assays in 95 cell types as part of ENCODE.^83^

### Colocalization of imQTLs with eQTLs:

To assess the overlap between imQTL interaction terms and expression quantitative trait loci (eQTLs), we performed colocalization analyses between imQTLs and previously published GTEx v8 eQTLs using the “coloc” R package (v5.2.3)^28^ with default priors (p_1_=1×10^−4^, p_2_=1×10^−4^, and p_12_=1×10^−5^). Colocalization was evaluated within the same tissue where imQTLs were identified, as well as across all tissues in which those imQTLs were observed (i.e. whole blood, colon, lung, ovary, and prostate). For each CpG-eGene pair with strong evidence of colocalization (posterior probability for shared signal, PP4 > 0.5), the top colocalized eQTL was defined as the one with the highest PP4 value. As a supplementary analysis, we utilized biologically informed priors; assuming 10 million common variants in the genome, with 10,000 variants regulating gene expression, 50,000 regulating DNAm, and 5,000 which regulate both gene expression and DNAm, we obtained priors of p_1_=5×10^−4^, p_2_=4.5×10^−3^, and p_12_=5×10^−4^.

### Colocalization of imQTLs with GWAS:

We additionally performed colocalization analyses between imQTL interaction terms and 90 GWAS health-related traits and diseases spanning a variety of phenotypes including aging, cancer, gastrointestinal disease, psychiatric-neurological disease, skeletal disease, and anthropometric, blood-related, cardiometabolic, immune-related, and morphological traits.^29–51^ We similarly used the “coloc” R package (v5.2.3)^28^ with default priors to perform imQTL-GWAS colocalization analyses. As a corollary analysis, we utilized biologically informed priors that assumed among 10 million common variants through the genome, 30,000 are involved in GWAS across any human health-related traits/diseases, 50,000 regulate variants DNAm, and 15,000 variants both increase susceptibility to health-related traits/diseases and regulate DNAm; these assumptions resulted in priors including p_1_=1.5×10^−3^, p_2_=3.5×10^−3^, and p_12_=1.5×10^−3^.

## Supplementary Material

Supplementary Files

This is a list of supplementary files associated with this preprint. Click to download.

- NatureGeneticsSupplementaryTables.xlsx
- NatureGeneticsSupplementaryFigures.docx

## Data Availability

The summary statistics for imQTL lead variants mapped in GTEx and HEALS will be uploaded to Zenodo. Full summary statistics for imQTLs mapped in each tissue/cell type combination require large memory for storage (~2.4 TB) and will be available upon request to the authors. All GTEx protected data are available via dbGaP (phs000424.v9); access to the DNAm raw data is provided through the AnVIL platform (https://anvil.terra.bio/#workspaces/anvil-datastorage/AnVIL_GTEx_V9_hg38). DNAm and genotyping data from HEALS is available on dbGaP with the accession code phs003839.v1.p1. The generic four-cell type deconvolution panel (*centEpiFibEndoIC.m*) will be uploaded to Zenodo and included as a reference panel in an update to the “EpiDISH” R package.

## CODE AVAILABILITY STATEMENT

All code developed to process DNAm and genetic data and all subsequent analyses are available at: https://github.com/james-li-projects/multi_tissue_celltype_imQTL.
